# Socioeconomic and behavioral factors of hypertension among Indian tribal population: Evidence from national family health survey 5

**DOI:** 10.1371/journal.pone.0312729

**Published:** 2024-12-27

**Authors:** Bodhi Sri Vidya Vennam, Sai Sushma Kuppli, Jayanta Kumar Bora, Soumya Swaroop Sahoo, Chaitanya Gujjarlapudi, Devi Madhavi Bhimarasetty, Ganga Nagamani Nerusu, Sonu Goel

**Affiliations:** 1 Department of Community Medicine, GITAM Institute of Medical Sciences and Research, Rushikonda, Visakhapatnam, Andhra Pradesh, India; 2 Department of Community Medicine, Andhra Medical College, Visakhapatnam, Andhra Pradesh, India; 3 VART Consulting, Mumbai, India; 4 Department of Community & Family Medicine, AIIMS, Bathinda, India; 5 Community Medicine, Government Medical College, Vizianagaram, Andhra Pradesh, India; 6 Adjuncant Associate Clinical Professor School of Medicine, University of Limerick, Limerick, Ireland; Institute for Social and Economic Change, INDIA

## Abstract

**Background:**

All Indian ethnic groups are experiencing an upsurge in the prevalence of hypertension. The objective of the present study was to explore the association between socioeconomic and behavioral factors of hypertension among the tribal population of India.

**Methods:**

We used the National Family Health Survey (NFHS) round 5 data conducted in 2019–2021. The final sample size was 67263 tribal women and 8441 tribal men aged 15 to 49 years. Chi-square test and the Cochran-Armitage trend test were employed to evaluate the association. Complex samples logistic regression analysis was conducted using clusters and sampling weights. Interstate variation of the prevalence of hypertension by gender was depicted with spatial maps.

**Results:**

The prevalence of hypertension among tribal women and men was 12% and 16·2%, respectively. All the independent variables were included in the multivariate model as all were significant at p<0.25 in bivariate analysis. Among both tribal women and men increasing age and at-risk waist hip ratio had higher likelihood ratios for hypertension. Among women, urban residence, lower education status, wealth status and consumption of alcohol were found to be significant predictors of hypertension. Among men, usage of smokeless tobacco was strongly associated with hypertension.

**Conclusion:**

Our study highlights a higher prevalence of hypertension found in older age, smokeless tobacco users, and abdominal obesity risk among the tribal population. There are interstate variations in the prevalence of hypertension among both men and women. These findings help in identifying the risk factors and geographical locations to be prioritized for hypertension prevention and control and in formulating health action plans focused on the tribal population in India. Appropriate intervention approaches need to be adopted to increase hypertension awareness and control practices, specifically focusing on tribals.

## Introduction

Hypertension is a chronic medical condition leading to mortality from cardiovascular and renal diseases. Globally, the number of adults with hypertension doubled from 650 million in 1990 to 1·3 billion in 2019. The prevalence of hypertension has declined in high-income countries but increased in the Western Pacific Region and the Southeast Asia Region (29% to 32%). About 78% of adults with hypertension live in low-and middle-income countries (LMICs) [1]. In LMICs, the prevalence of hypertension increased between 1990 and 2020 in urban and rural areas, contrary to the previous belief that hypertension is an urban phenomenon (pooled prevalence 30·5% and 27·9% in urban and rural areas, respectively) [2]. The previous round of the NFHS (NFHS 4, 2015–16) reported the age-adjusted prevalence of hypertension as 11·3% in the general population in the 15–49 age group [3]. Studies done in India based on the NFHS-4 have reported a higher prevalence of hypertension among men compared to women [1,3]. There is a wide variation in the proportion of hypertensives across various Indian states [3].

An increase in blood pressure is a result of multiple factors. The main contributors are a high salt diet, physical inactivity, overweight and obesity, alcohol consumption, and usage of tobacco [1].

India is the most populous country in the world, with a rich cultural and ethnic diversity and is home to the most varied and extensive tribal population [4]. The tribal population constitutes nearly 8·6% of the Indian population [5]. They are scattered all over the country and remain a socioeconomically disadvantaged group with morbidity and mortality indicators higher than the national average and a lack of pertinent access to healthcare. A meta-analysis of twenty studies conducted from 1981 to 2011 reported the pooled prevalence of hypertension as 16·1% in tribals. It also reported the prevalence of hypertension in the tribal population (22·5%) to be lower than that of the general population (32·5%) for the comparable periods (2001–11). Hypertension prevalence has been reportedly lower among tribal women than in tribal men [6]. Studies conducted across India have reported a higher prevalence of tobacco usage and hypertension among the tribal population [7–9]. According to a survey done among Kashmiri tribes, tribal men (46·7%) showed a higher prevalence of hypertension than women (37·9%) [10]. The blood pressure of tribal populations results from the interactions between contrasting forces of a protective traditional lifestyle (frugal diet and high physical activity) and factors that increase vulnerability, such as high substance use and increasing embracement of modern lifestyle patterns [11].

The burden of hypertension in India is constantly on the rise due to changes in lifestyle and behavioral factors brought in by a socioeconomic and epidemiological transition [12]. The tribal population, which constitutes nearly a tenth, is also gradually experiencing changes. Due to diverse ethnic backgrounds, diets, behavioral habits, and geographical distribution, tribal populations are expected to have community-specific risk factors [13]. Low levels of literacy, modest socioeconomic status, and limited access to healthcare make this population more vulnerable.

As per our knowledge, there is a paucity of studies on the prevalence of hypertension and its associated factors among tribal populations in India. The present study intends to explore the association between socioeconomic and behavioral factors of hypertension and to provide disaggregated data about them on a nationally representative tribal population using the recently available NFHS 5 data.

The results of this study are expected to generate evidence to guide health policy focusing on devising targeted interventions for hypertension prevention and control among this socioeconomically disadvantaged population.

## Data and methods

In this study, we used the recently available nationally representative NFHS-5 data (2019–21), a large-scale multi-round survey conducted throughout India. The International Institute for Population Sciences conducts it, the nodal agency under the stewardship of the Ministry of Health and Family Welfare, Government of India (GOI). The NFHS fifth-round survey provides information on 636,699 households, 699476 women (age 15–49 years), and 101,839 men (age 15–54 years) [14].

**Fig 1:**
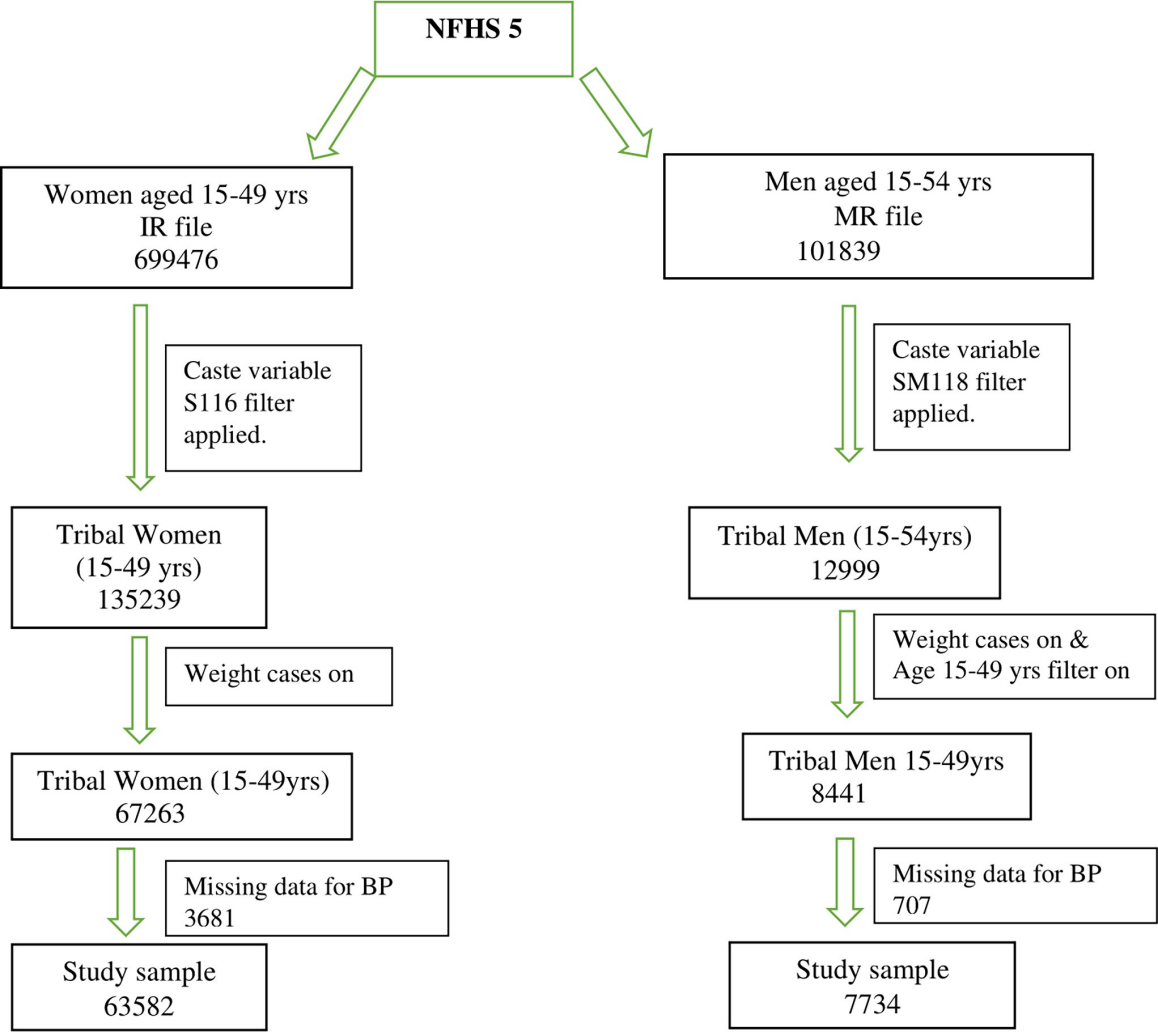
Sample selection process for the study analysis.

The NFHS survey follows a two-stage sampling approach for selecting villages and census-enumeration blocks (CEBs) in rural and urban areas as primary sampling units (PSUs). The 2011 Census data was used as the sampling frame. The final sample PSUs and CEBs were selected using Probability Proportionate to size sampling. The survey design and sample details can be accessed in the national report [14,15]. In our study, the final sample size for tribal women (67,263) and men (8441) aged 15–49 years was used from the individual level data sets Individual Recode (IR) file and Male Recode (MR) file, respectively, in our analysis [14]. The inclusion and exclusion criteria for the final sample size are presented in Fig 1.

### Operational definitions

#### Hypertension

From the three blood pressure readings taken in each subject at a gap of 5 min, the average of the last two readings was used for the analysis. Average systolic blood pressure of ≥140 mm Hg (or) and average diastolic blood pressure of ≥90 mm Hg (or) currently taking a prescribed medication to lower blood pressure were considered to have hypertension in the present study [14,15].

#### Explanatory variables

The variables included were age groups (15–24,25–34,35–44 and 45–49), religion (Hindu, Muslim, Christian, others), level of education (no education, primary, secondary and higher), type of residence (urban or rural), marital status (married, single/unmarried), wealth index (poorest, poorer, middle, richer and richest), alcohol usage (yes/no), fruit consumption (never, occasionally, weekly and daily), smoking tobacco use (yes/no) and smokeless tobacco use (yes/no).

Since demographic performance in India differs by region of residence, special consideration has been given to adjusting estimates for the region of residence. For this purpose, India was divided into six regions in NFHS surveys based on geographical locations with similar cultural backgrounds. The six regions are the north region (Jammu and Kashmir, Himachal Pradesh, Punjab, Chandigarh, Uttarakhand, Haryana, Delhi), the central region (Rajasthan, Uttar Pradesh, Chhattisgarh, and Madhya Pradesh); the east region (West Bengal, Jharkhand, Odisha, and Bihar); the north-East region (Sikkim, Arunachal Pradesh, Nagaland, Manipur, Mizoram, Tripura, Meghalaya, and Assam); the west region (Gujarat, Maharashtra, Goa, Dadra & Nagar Haveli and Daman & Diu); and the south region (Andhra Pradesh, Karnataka, Kerala, Tamil Nadu, and Puducherry).

Waist-hip ratio (abdominal obesity) was categorized as normal < 0·85 (women) and < 0·90 (men) and at risk if ≥0·85 (women) and ≥ 0·90 (men) [15,16]. A person who smokes cigarettes/pipes/cigars/bidis/hookah is labelled as a ‘tobacco smoker’, and a person who chewed tobacco in the form of khaini/gutkha/paan masala-tobacco/paan-tobacco is called a ‘smokeless tobacco user’.

### Statistical analysis

The analysis was performed using the SPSS 25 version (IBM SPSS Statistics for Windows, Version 25.0. Armonk, NY: IBM Corp), and the maps were generated using the QGIS software (version 3.36.3), a free and open-source geographic information system (GIS) application. It’s licensed under the GNU General Public License (GPL), which allows users to inspect and modify the source code. QGIS is owned by its contributors and is hosted on https://www.qgis.org. Geographical materials used to create the map, such as shapefiles, were sourced from https://onlinemaps.surveyofindia.gov.in/. The authors have designated these figures under the Creative Commons Attribution 4.0 (CC BY 4.0) license. The current study aims to investigate the socioeconomic and behavioral determinants of hypertension (HTN) among the Indian tribal population, using data from the National Family Health Survey 5 (NFHS-5). The dependent variable in this analysis is the presence of hypertension (HTN), which is binary. The independent variables include Age (ordinal), Religion (nominal), Education (ordinal), Residence (nominal), Region (nominal), Marital Status (nominal), Wealth Index (ordinal), Alcohol Consumption (nominal), Fruit Consumption (ordinal), Waist-Hip Ratio (WHR) (nominal), Smoking Tobacco (nominal), and Smokeless Tobacco (nominal). The analysis is stratified by gender, and weighted analysis was conducted using the weights suggested by NFHS to ensure representativeness and accuracy of the results.

#### Bivariate analysis

For the bivariate analysis, the Chi-square test and the Cochran-Armitage trend test were employed to evaluate the association between each independent variable and hypertension at a 5% level of significance (LOS).

#### Multivariate analysis

In the bivariate analysis, all variables for both men and women had p-values below 0.25, and were therefore included in the final multivariate model. Complex samples logistic regression analysis was conducted using clusters and sampling weights. Adjusted odds ratios (AOR) with 95% confidence intervals (CI) were reported. Global p-values were obtained using the Wald test, with p-values less than 0.05 considered statistically significant.

### Ethical statement

The study is based on an anonymous, publicly accessible dataset, with no recognizable information about the survey participants, there is no need for an ethics statement for this research work.

## Results

Table 1 shows the distribution of socioeconomic and behavioral factors by the prevalence of hypertension among tribal women and men aged 15–49 years.

**Table 1:** Distribution of socioeconomic and behavioral factors by the prevalence of hypertension among tribal women and men aged 15–49 years, 2019–21, India.

		Prevalence of Hypertension				
	Tribal women^#^			Tribal men^#^		
	Yes n (%)	No n (%)	P value	Yes n (%)	No n (%)	P value
**Age groups (in years)**			**0.000**			**<0.001**
15–24	987 (4·5)	20764 (95·5)		161 (6·2)	2416 (93·8)	
25–34	1856 (9·5)	17766 (90·5)		368 (15·9)	1953 (84·1)	
35–44	2877 (18·8)	12444 (81·2)		460 (23·3)	1515 (76·7)	
45–49	1889 (27·4)	4999(72·6)		265 (30·8)	594 (69·2)	
**Religion**			0.07			0.12
Hindu	6504(11·9)	48218(88·1)		1045 (16)	5493 (84)	
Muslim	145 (11)	1176(89)		23 (12)	168 (88)	
Christian	672 (13)	4493 (87)		152 (19·8)	616 (80·2)	
Others	287 (12·1)	2086 (87·9)		35 (14·8)	202 (85·2)	
**Education**			**<0.001**			**<0.001**
No education	3628 (16·4)	18442 (83·6)		261 (19·7)	1064 (80·3)	
Primary	1090 (13)	7326 (87)		207 (17·8)	957 (82·2)	
Secondary	2471 (8·7)	25829 (91·3)		658 (15)	3743 (85)	
Higher	420 (8·8)	4375 (91·2)		128 (15·2)	714 (84·8)	
**Residence**			**<0.001**			**<0.001**
Urban	1272 (14·2)	7698 (85·8)		242 (19·4)	1006 (80·6)	
Rural	6337 (11·6)	48274 (88·4)		1012 (15·6)	5472 (84·4)	
**Region**			**<0.001**			**<0.001**
South	1040 (13·9)	6462 (86·1)		237 (19·9)	955 (80·1)	
North	524 (8)	6002 (92)		54 (10·8)	444 (89·2)	
Central	1829 (12·5)	12824 (87·5)		145 (17·8)	668 (82·2)	
East	1815 (11·4)	14045 (88·6)		230 (14·6)	1345 (85·4)	
West	1518 (11·8)	11350 (88·2)		375 (14·9)	2139 (85·1)	
Northeast	882 (14·3)	5290 (85·7)		213 (18·7)	927 (81·3)	
**Marital status**			**<0.001**			**<0.001**
Married	6116 (13·4)	39467 (86·6)		991 (20·3)	3880 (79·7)	
Unmarried / Single	1492 (8·3)	16505 (91·7)		263 (9·2)	2598 (90·8)	
**Wealth Index**			**<0.001**			**<0.001**
Poorest	3174 (11·5)	24542 (88·5)		443 (14·7)	2572 (85·3)	
Poorer	1882 (11·6)	14314 (88·4)		339 (16·4)	1725 (83·6)	
Middle	1220 (12)	8960 (88)		233 (16·9)	1143 (83·1)	
Richer	797 (13·4)	5139 (86·6)		127 (15·7)	682 (84·3)	
Richest	535 (15·1)	3017 (84·9)		111(23·8)	356 (76·2)	
**Drink alcohol**			**<0.001**			**<0.001**
Yes	528 (21)	1981(79)		537 (20·5)	2077 (79·5)	
No	7080 (11·6)	53991 (88·4)		717 (14)	4401 (86)	
**Eating fruits**			0.09			0.24
Never	202 (12·2)	1455 (87·8)		43 (26·1)	122 (73·9)	
Occasionally	4487 (11·7)	33806 (88·3)		597 (14·9)	3413 (85·1)	
Weekly	2423 (12·3)	17325 (87·7)		520 (17·3)	2487 (82·7)	
Daily	497 (12·8)	3386 (87·2)		93 (16·9)	457 (83·1)	
**Waist hip ratio†**			**<0.001**			**<0.001**
At risk	4449 (13·1)	29593 (86·9)		661 (21·3)	2440 (78·7)	
Normal	3141 (10·7)	26286 (89·3)		591 (12·8)	4031 (87·2)	
**Smoking tobacco**			**<0.001**			**<0.001**
Yes	205 (17·2)	990 (82·8)		359 (19·1)	1517 (80·9)	
No	7404 (11·9)	54983 (88·1)		895 (15·3)	4962 (84·7)	
**Smokeless tobacco**			**<0.001**			**<0.001**
Yes	1049 (17·1)	5071 (82·9)		581 (19·7)	2366 (80·3)	
No	6559 (11·4)	50902 (88·6)		673 (14·1)	4112 (85·9)	
**Total**	**7609 (12)**	**55973 (88)**		**1255 (16·2)**	**6479 (83·8)**	

#Missing values were excluded, in both the groups, in bivariate analysis.

**†**Waist hip ratio -At risk: ≥0·85 women & ≥0·90 men, Normal: <0·85 women, and <0·90 men.

**In tribal women,** the prevalence of hypertension was 12%. Hypertension prevalence steadily increased with age; the highest prevalence was observed among the age group 45–49 years (27·4%). The prevalence of hypertension increased at lower levels of education, with the highest prevalence observed in tribal women with no education (16·4%). A higher prevalence of hypertension was reported among urban tribal women (14·2%) in comparison with rural counterparts (11·6%). In terms of geographical distribution, tribal women in the Northeast region (14·3%) showed a significantly higher proportion of hypertension. Married tribal women (13·4%) reported a higher prevalence of hypertension than unmarried/single women. The richest wealth index (15·1%) showed a higher proportion of hypertensives. Alcohol use (21%), Abdominal obesity (13·1%), Smoking tobacco (17·2%), and Smokeless tobacco use (17·1%) were found to be significantly associated with hypertension among tribal women. Among the females who were found to have hypertension (HTN), 17.4% were receiving treatment, and 0.7% had uncontrolled hypertension.

In tribal men, the prevalence of hypertension was 16·2%. Hypertension prevalence steadily increased with age and was highest among the age group 45–49 years (30·8%). An increased prevalence of hypertension was also observed among tribal men (19·7%) with no education. Tribal men in an urban area (19·4%) reported a higher prevalence of hypertension than their rural counterparts. In terms of geographical distribution, tribal men in the Southern region (19·9%) showed a significantly higher proportion of hypertension. Married tribal men (20·3%) reported a higher prevalence of hypertension than unmarried/single men. The higher proportion of hypertensives was seen from the richest wealth index (23·8%). Alcohol use (20·5%), abdominal obesity (21·3%), smoking tobacco (19·1%), and smokeless tobacco use (19·7%) were found to be significantly associated with hypertension among tribal populations. The results indicated that all variables except religion and consumption of fruits were significantly associated with hypertension in both tribal women and men at the 5% level of significance. Among the males who were found to have hypertension (HTN), 9.6% were receiving treatment, and 0.6% had uncontrolled hypertension.

Table 2 presents the association of socioeconomic and behavioral factors with hypertension among tribal women and men aged 15–49 years.

**Table 2:** Association of socioeconomic and behavioral factors with hypertension among tribal women and men aged 15–49 years, 2019–21, India.

	Tribal women		Tribal men	
	Adjusted OR (95% CI)	P-value	Adjusted OR (95% CI)	P-value
**Age groups (in years)**		<0.001		<0.001
**15–24 (Ref)**				
**25–34**	2.13 (1.91–2.36)		2.41 (1.75–3.31)	
**35–44**	4.44 (3.99–4.95)		3.84 (2.50–5.91)	
**45–49**	7.05 (6.24–7.96)		5.52 (3.52–8.66)	
**Religion**		0.95		0.11
**Hindu (Ref)**				
**Muslim**	0.97 (0.78–1.21)		1.61 (0.72–3.57)	
**Christian**	0.97 (0.74–1.13)		1.21 (0.52–2.82)	
**Others**	0.94 (0.74–0.12)		1.06 (0.38–2.95)	
**Education**		**<0.01**		0.8
**No education**	1.32 (1.11–1.58)		0.97 (0.58–1.64)	
**Primary**	1.27 (1.05–1.53)		0.95 (0.62–1.48)	
**Secondary**	1.12 (0.95–1.33)		1.06 (0.73–1.53)	
**Higher (Ref)**				
**Residence**		**0.04**		0.32
**Urban**	1.13 (1.01–1.27)		1.16 (0.87–1.54)	
**Rural (Ref)**				
**Region**		**<0.001**		**0.005**
**South (Ref)**				
**North**	0.93 (0.81–1.08)		0.54 (0.37–0.80)	
**Central**	0.92 (0.79–1.08)		0.90 (0.65–1.25)	
**East**	0.62 (0.52–0.73)		0.63 (0.42–0.97)	
**West**	1.06 (0.93–1.21)		0.68 (0.46–0.99)	
**Northeast**	1.11 (0.96–1.29)		0.78 (0.54–1.13)	
**Marital Status**		0.06		0.74
**Married**	0.92 (0.84–1.00)		1.06 (0.75–1.50)	
**Unmarried / Single (Ref)**				
**Wealth Index**		**0.005**		0.54
**Poorest (Ref)**				
**Poorer**	0.87 (0.73–1.04)		0.57 (0.27–1.17)	
**Middle**	0.79 (0.66–0.94)		0.65 (0.31–1.37)	
**Richer**	0.77 (0.65–0.91)		0.66 (0.32–1.36)	
**Richest**	0.73 (0.61–0.87)		0.60 (0.28–1.29)	
**Drink alcohol**		**<0.001**		0.39
**Yes**	1.32 (1.18–1.48)		1.09 (0.90–1.32)	
**No (Ref)**				
**Eating Fruits**		0.21		0.12
**Never**	0.91 (0.80–1.04)		1.81 (0.86–3.80)	
**Occasionally**	0.87 (0.69–1.08)		0.93 (0.62–1.38)	
**Weekly**	0.97 (0.85–1.11)		1.08 (0.72–1.64)	
**Dailly (Ref)**				
Waist hip ratio†		**<0.001**		**<0.001**
**At Risk**	1.18 (1.10–1.29)		1.56 (1.29–1.89)	
**Normal (Ref)**				
**Smoking tobacco**		0.7		0.97
**Yes**	1.04 (0.86–1.25)		0.99 (0.80–1.24)	
**No (Ref)**				
**Smokeless tobacco**		0.08		**<0.001**
**Yes**	1.08 (0.99–1.18)		1.40 (1.15–1.69)	
**No (Ref)**				

Note: Ref- Reference category; OR- odds ratio.

**†**Waist hip ratio -At risk: ≥0·85 women & ≥0·90 men, Normal: <0·85 women, and <0·90 men.

Among tribal women, age was a significant factor in predicting hypertension (p<0.001). Women aged 25–34 had 2.13 times higher odds of having hypertension (AOR: 2.13, 95% CI: 1.91–2.36) compared to the reference group (15–24). The odds increased further for women aged 35–44 (AOR: 4.44, 95% CI: 3.99–4.95) and 45–49 (AOR: 7.05, 95% CI: 6.24–7.96). Education also played a role (p<0.01), with women having no education (AOR: 1.32, 95% CI: 1.11–1.58) and primary education (AOR: 1.27, 95% CI: 1.05–1.53) showing higher odds of hypertension. Urban residence was associated with higher odds of hypertension (AOR: 1.13, 95% CI: 1.01–1.27, p = 0.04), while region had a significant influence (p<0.001), with women from the East having reduced odds (AOR: 0.62, 95% CI: 0.52–0.73). Wealth index (p = 0.005) showed that women in the richest group had lower odds of hypertension (AOR: 0.73, 95% CI: 0.61–0.87). Alcohol consumption significantly increased the odds (AOR: 1.32, 95% CI: 1.18–1.48, p<0.001), and women at risk based on waist-hip ratio (WHR) also had higher odds of hypertension (AOR: 1.18, 95% CI: 1.10–1.29, p<0.001).

For tribal men, age was similarly a strong predictor of hypertension (p<0.001). The odds of hypertension increased with age, with men aged 25–34 having 2.41 times higher odds (AOR: 2.41, 95% CI: 1.75–3.31), 35–44 having 3.84 times higher odds (AOR: 3.84, 95% CI: 2.50–5.91), and 45–49 having 5.52 times higher odds (AOR: 5.52, 95% CI: 3.52–8.66) compared to the reference group. Regional differences were also significant (p = 0.005), with men from the North (AOR: 0.54, 95% CI: 0.37–0.80) and East (AOR: 0.63, 95% CI: 0.42–0.97) having lower odds of hypertension. Waist-hip ratio was a significant factor (p<0.001), with men at risk based on WHR having 1.56 times higher odds (AOR: 1.56, 95% CI: 1.29–1.89) of hypertension. Smokeless tobacco use also increased the odds of hypertension in men (AOR: 1.40, 95% CI: 1.15–1.69, p<0.001). However, other variables such as education, alcohol consumption, and wealth index did not show significant associations with hypertension.

### State-wise hypertension status among the tribal population

**Fig 2:**
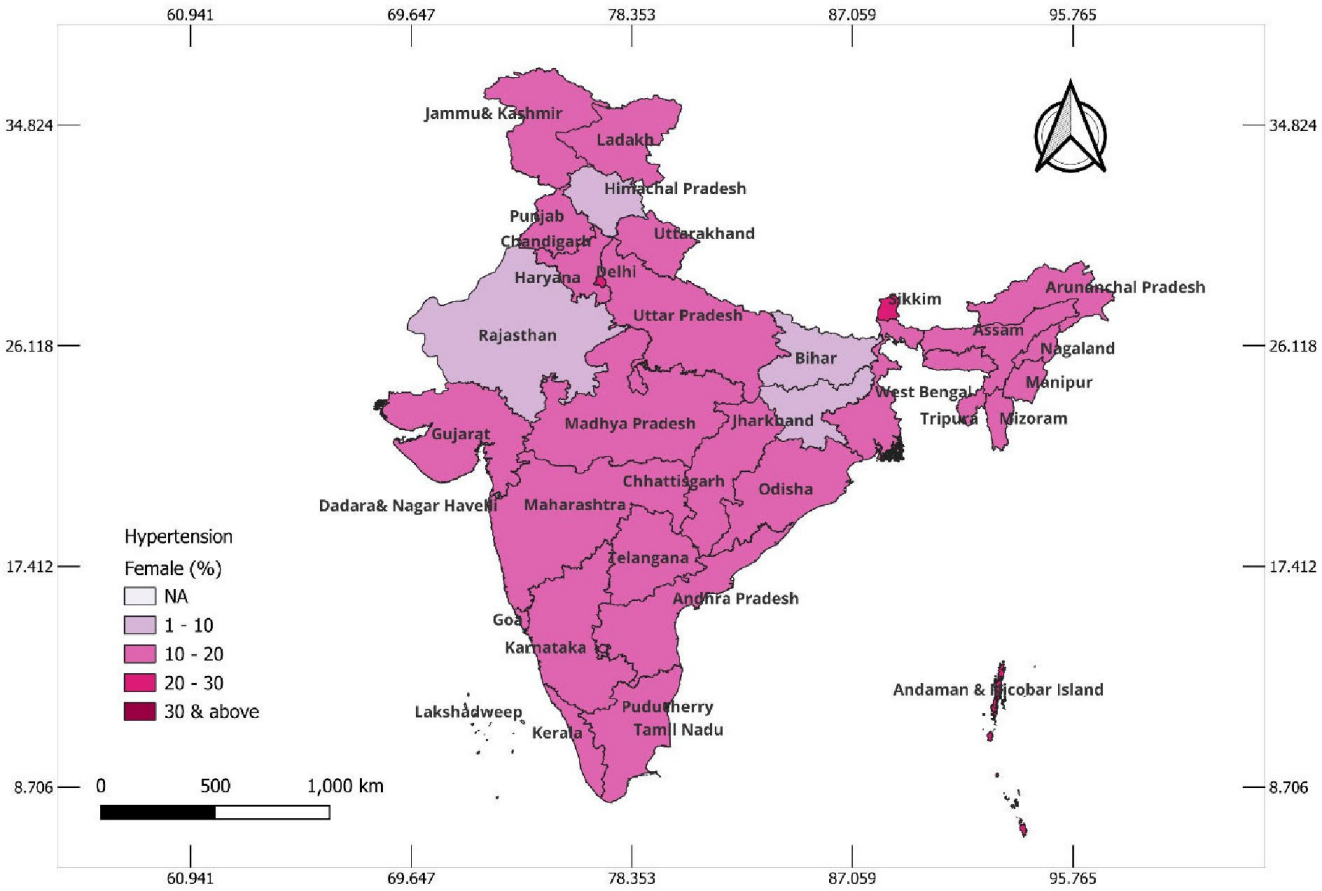
Map showing the prevalence of hypertension among tribal women aged 15–49 years in Indian states, 2019–21, India.

**Fig 3:**
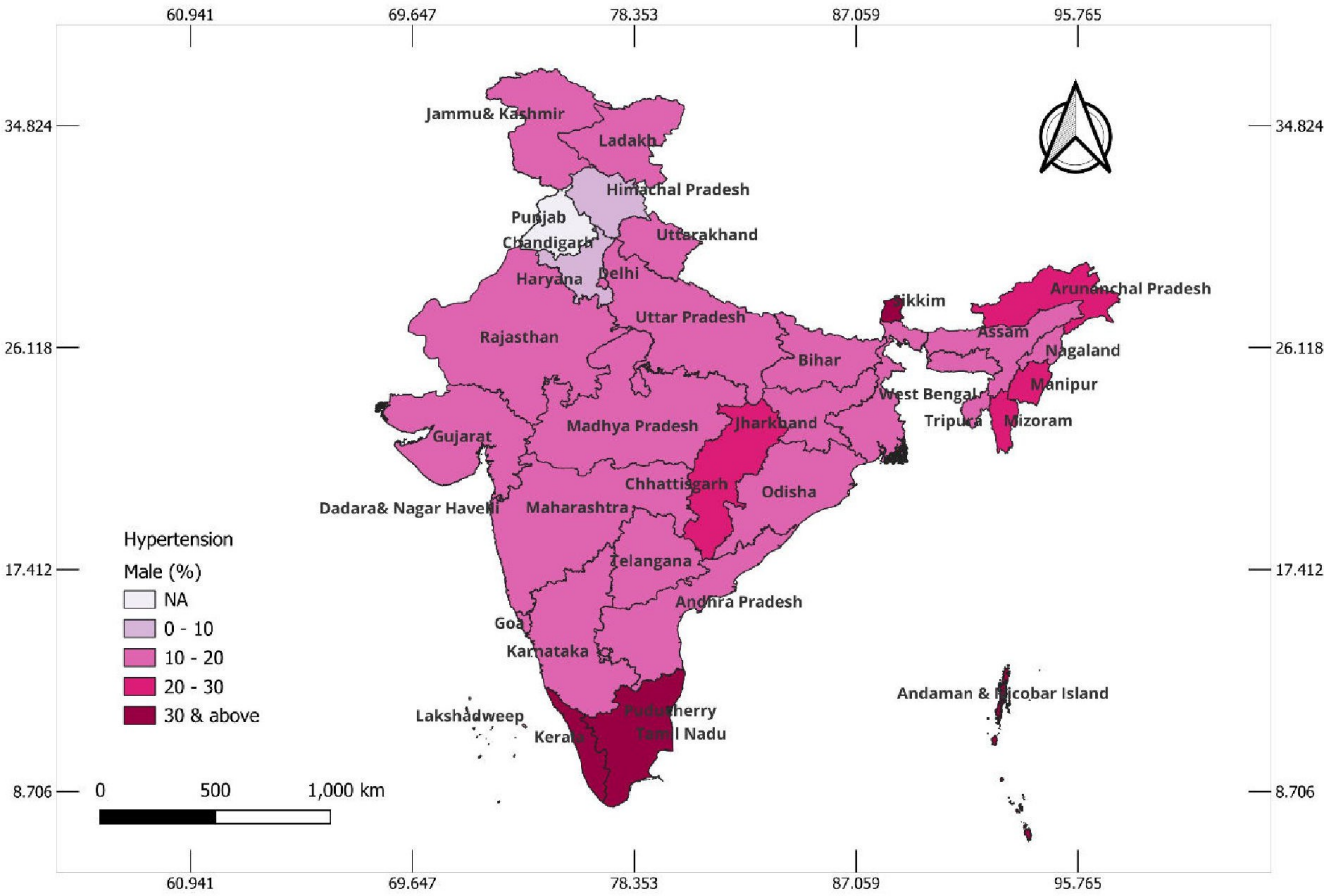
Map showing the prevalence of hypertension among tribal men aged 15–49 years in Indian states, 2019–21, India.

The state-wise prevalence of hypertension among tribal women and men is presented in Figs 2 & 3, respectively. Interestingly, in Fig 2, it can be observed that in most of the states, the prevalence of hypertension was higher among tribal men than women. Among the tribal women, the prevalence of hypertension was higher in the northern states of Punjab, Haryana, and Delhi; Sikkim and Arunachal Pradesh from the northeastern region; Goa from the western region; and Kerala, Tamil Nadu, Telangana, and Andaman & Nicobar Islands from the southern region. Fig 3 depicts a high prevalence of hypertension among tribal men from the northeastern states of Sikkim, Arunachal Pradesh, Manipur, Mizoram, and Tripura; Chhattisgarh from the central region; and Lakshadweep, Kerala, Tamil Nadu, Telangana, and Andaman & Nicobar Islands from the southern parts of the country.

## Discussion

Our study summarizes the prevalence of hypertension and its socioeconomic and behavioral factors among the tribal population using a nationally representative dataset of NFHS 5. India is going through an epidemiological transition, and the tribal population too is not immune to it. With gradual acculturation, the tribal population hasn’t remained unaltered from the factors driving the hypertension epidemic in the general Indian population [6,13,17,18]. Hence addressing these factors needs the utmost attention. The prevalence of hypertension in 15–49-year age group tribal men (16·2%) and women (12%) was lower than the national average (men 24%, women 21%) reported in NFHS 5 survey reports. Also, a study by Kusuma YS et al in Odisha reported a higher prevalence of 24·8% in men and 13·4% in women tribal population [19]. In the current study, a high prevalence of hypertension was reported in tribal women of Andaman and Nicobar Islands (29·2%), Sikkim (25·3%), and tribal men of Sikkim (36·2%) and Tamil Nadu (35·1%). Other studies conducted among tribal people in Assam [20], Nicobar tribes [10], and Native Americans [21] reported a higher prevalence of hypertension when compared to the present study. The reason might be that we analyzed a national-level dataset representing tribal populations across the country, whereas most of these studies were conducted in specific states, confined to geographical boundaries. Moreover, this can be attributed to lower awareness levels for hypertension, in addition to hardships of geographical location and inadequate access to health care. Acculturation to accelerated environmental changes and the ability to adapt also contribute to the growing prevalence of hypertension in the tribal population.

The proportion of hypertensives increased with age, with a noticeable increase in the 45–49 age group. This has been reported by other studies in the tribal population. [7–9,22,23]. Age is an independent risk factor for hypertension, irrespective of ethnicity.

Tribal men and women with low levels of education reported an increased prevalence of hypertension. This is in accordance with other studies that show that higher levels of education act as a protective factor because of better health literacy and health-seeking behavior [24].

The prevalence was higher in urban areas than in rural areas. This is on the expected lines in the urban tribal population due to the gradual acculturation and embracement of urbanization and changing lifestyles, moving away from the traditional frugal diets and physical activity. This urban-rural difference has also been highlighted in other studies [25–27].

In the present study among hypertensives, the smoking habit was present in 17·2% of the tribal women and 19·1% of the tribal men. The proportion of hypertensives was similar in those using smokeless tobacco (women 17·1%, men 19·7%). All types of tobacco use (smoke and smokeless) had a significant association with hypertension in the tribal population. In the present study, among different forms of tobacco users, tribal men had a marginally higher prevalence of hypertension than tribal women. Similar results have been reported in studies conducted among other tribal populations [10]. Tobacco abuse has been reported to be a significant determinant of hypertension in earlier studies done using NFHS-4 data [28]. In India, tobacco is not merely a socio-cultural problem, but multifaceted with geopolitical, biomedical, economic, and acculturation aspects [29]. This variation can be attributed to ingrained social beliefs and cultural practices in the tribal population. Also, in many states like Chhattisgarh, Bihar, and Madhya Pradesh, a large portion of the tribal population, particularly females are involved in the tobacco industry (beedi making) providing a source of livelihood [30]. Higher tobacco usage has been reported in studies done among tribals in Assam and West Bengal [20,31]. A recent secondary data analysis from the Longitudinal Ageing Study in India dataset by Murmu J et al also revealed a higher prevalence of tobacco use at a high of 46% [32]. Lack of awareness, family environment, low educational status, and easy access to tobacco products play an important role in tobacco consumption in tribal areas. In some tribal groups, it is also considered a means to concentrate on work [33]. Tribal communities live in geographical isolation, in poor conditions, have a poor literacy rate and their traditional occupations make them more vulnerable to the use of tobacco products, chronic use of which is an independent risk factor for hypertension. Use of smokeless tobacco was found to have higher odds of being hypertensive among tribal men. Many studies have demonstrated a proven risk of developing hypertension with chronic smokeless tobacco use [34,35]. However, as the NFHS 5 data was generated from cross-sectional survey, the chronicity of exposure cannot be determined.

In the present study, abdominal obesity was determined from Waist-hip ratio. A higher waist-hip ratio is a surrogate marker of obesity which is a contributory factor for hypertension. Our findings suggest that the tribal population with abdominal obesity had a higher risk of being hypertensive in both men and women. Similar epidemiological studies have also reported a higher prevalence of hypertension in tribal populations with abdominal obesity [25,36,37].

Among both tribal women and men, the wealth index has not shown any significant association with hypertension. Laxmaiah et al. did not report any significant association between socioeconomic status and hypertension in their study [9]. Few other studies have reported an inverse association between hypertension and socioeconomic status [23,24].

In the current study, hypertension was found to be higher among females in the northern states and some northeastern states like Sikkim and Arunachal Pradesh and among males from Northeastern states, Tamil Nadu and Kerala. A similar finding has been reported in a study conducted among tribals in Nagaland [38]. The higher prevalence in the northeast in both the genders may be attributed to ethnicity and food habits [39]. The factors for the southern states of Kerala and Tamil Nadu may be an advanced stage of epidemiological transition with increasing urbanization, changing dietary habits, growing obesity, and social stress [3].

### Strengths and limitations of the study

The strength of the study is a nationally representative tribal dataset with an adequate sample size. It helped us to provide estimates of the prevalence of hypertension across geographical regions and its association with different sociodemographic factors influencing hypertension. However, our study has a few limitations. The data is cross-sectional, so it does not help us explore causal pathways underlying the reported associations. The self-reporting of behavioral factors like alcohol and tobacco consumption may lead to social desirability bias. This may have underestimated the proportion of actual use in the population, leading to a lower attributable risk of these contributory risk factors. Additionally, tribals consume foods like wild tubers and flowers, and several cultural behavior-related indicators unique to the tribal population like environmental and occupational risk factors, were not included in our analysis due to non-availability in the NFHS data. Follow-up studies to explore the varied risk factors of hypertension including behavioral and social factors, need to be undertaken to provide holistic insights into the causative factors, trends, and long-term effects among these ethnically diverse tribal populations. The data collection for our study coincided with the COVID-19 pandemic, which may have influenced the behaviors and health outcomes of the participants. Changes in lifestyle, such as increased stress, altered physical activity patterns, and changes in dietary habits due to lockdowns and other restrictions, could have impacted blood pressure levels. These factors introduce variability that could affect the generalizability of our findings. Therefore, the results should be interpreted with caution, considering the potential impact of the pandemic on the participants’ behaviors and health outcomes.

## Conclusion

Our study highlights a higher prevalence of hypertension along with the socioeconomic and behavioral factors in the tribal population. The tribal women from northern states and northeastern states like Sikkim and Arunachal Pradesh and the southern states of Kerala, Tamil Nadu, and Telangana were found to have a higher prevalence of hypertension. The tribal men from Northeastern states, Chhattisgarh, Kerala, and Tamil Nadu, were found to have a higher prevalence of hypertension. As a part of the tribal health policy of the Government of India, these states should be prioritized for hypertension control in health action plans with emphasis on preventive measures and modifiable risk factors. There is a need to set up population-based cohorts in tribal populations to prioritize targeted approaches for controlling hypertension. The findings of our study would appraise policymakers of the growing burden of non-communicable disease and its risk factors in vulnerable tribal populations. Common significant factors for both men and women were age, waist-hip ratio, and regional differences, with older age and an at-risk waist-hip ratio increasing the likelihood of hypertension. Additionally, distinct factors emerged for each gender. Among women, urban residence, lower education levels, alcohol consumption, and wealth status were significant predictors. Among men, smokeless tobacco use was strongly associated with hypertension. These results highlight common and gender-specific risk factors, emphasizing the need for tailored public health interventions addressing these disparities in tribal communities.
